# Emergency department contribution to HCV elimination in the Iberian Peninsula

**DOI:** 10.1186/s12245-023-00570-5

**Published:** 2024-01-04

**Authors:** Maria Buti, Inês Vaz-Pinto, Vítor Magno Pereira, Marta Casado, Jordi Llaneras, Ana Barreira, Catarina Esteves, Mafalda Guimarães, Ana Gorgulho, Tomás Mourão, Elisa Xavier, Luís Jasmins, Ana Paula Reis, Nancy Faria, Bruno Freitas, Graça Andrade, Anny Camelo-Castillo, Manuel Ángel Rodríguez-Maresca, Alba Carrodeguas, Diogo Medina, Rafael Esteban

**Affiliations:** 1 Hospital Universitari Vall d’Hebron and CIBEREHD del Instituto Carlos III, 119, 08035 Barcelona, Spain; 2 Hospital Dr. José de Almeida, Cascais, Portugal; 3 Hospital Dr. Nélio Mendonça, Madeira, Portugal; 4 Hospital Universitario Torrecárdenas, Almería, Spain; 5 Hospital Universitari Vall d’Hebron, Barcelona, Spain; 6 Gilead Sciences, Madrid, Lisbon Spain

**Keywords:** HCV, Emergency department, Screening, Diagnosis

## Abstract

**Background:**

Undiagnosed cases of hepatitis C virus (HCV) infection result in significant morbidity and mortality, further transmission, and increased public health costs. Testing in emergency departments (EDs) is an opportunity to expand HCV screening. The goal of this project was to increase the proportion of eligible patients screened for HCV in urban areas.

**Methods:**

An opportunistic automated HCV screening program was implemented in the EDs of 4 public hospitals in Spain and Portugal at different periods between 2018 and 2023. HCV prevalence was prospectively evaluated, and single-step or reflex testing was used for confirmation in the same sample.

**Results:**

More than 90% of the population eligible for testing were screened in the participating centers. We found HCV antibody seroprevalence rates ranging from 0.6 to 3.9%, with between 19 and 53% of viremic individuals.

**Conclusions:**

Opportunistic HCV screening in EDs is feasible, does not disrupt ED activities, is highly effective in increasing diagnosis, and contributes to WHO’s HCV elimination goals.

## Introduction

An estimated 58 million people have chronic hepatitis C infection (HCV), and about 1.5 million new infections are diagnosed annually worldwide [1]. The World Health Organization (WHO) estimated that in 2019, approximately 290,000 people died from hepatitis C, mostly as a result of cirrhosis and hepatocellular carcinoma (primary liver cancer) [1]. As a part of the first global health sector strategy on viral hepatitis (2016–2021), the WHO aims to eliminate viral hepatitis as a public health problem by reducing new viral hepatitis infections by 90% and reducing viral hepatitis deaths by 65% by 2030 [1]. Reaching these objectives will require a substantial increase in the number of HCV screenings performed to diagnose and treat 80% of the estimated 58 million HCV-infected persons [1]. However, by 2017, only about 20% of this population were aware of their infection, and only 5 million individuals diagnosed with HCV had received appropriate therapy [2]. In fact, an estimated 22,500 and 12,300 people living with chronic HCV in Spain and Portugal are unaware of their infection [3]. Researchers have projected the timelines for selected countries to achieve HCV elimination, with Spain initially predicted to be among the first nations globally to reach these goals well before 2030 [4]. However, the advent of the COVID-19 pandemic seems to have deferred this milestone [5]. Meanwhile, with current policies, Portugal is unlikely to achieve elimination before 2050 [6].

Although generally held by health systems as their strategy of choice, risk-based or targeted screening is insufficiently implemented, resulting in significant levels of missed opportunities for diagnosis [7,8]. Underdiagnosis and late diagnosis of HCV infection have 3 main consequences: (1) worse prognosis and loss of quality of life in patients with late diagnosis, (2) increased healthcare costs, and (3) greater spread of the infection. More effective and evidence-based models of HCV preventive care are needed.

The European Center for Disease Prevention and Control (ECDC) is promoting an increase in test coverage and uptake, especially for those most at risk, as an essential element of any strategy to eliminate HIV, HBV, and HCV in the European Union and European Economic Area (EU/EEA) [9]. In line with this, many screening procedures have been developed in recent years with the goal of finding previously undiagnosed HCV carriers in populations with risk factors for HCV infection, which is regarded as an essential strategy and a minimal best practice [10]. However, the outcomes gained with this method are not always as good as anticipated [11–13]. Urban EDs are well-positioned for detecting HCV infection and might be suitable places to reach patient profiles that could be difficult to access at other levels of care [14].

The aim of our project was to increase the proportion of eligible patients for HCV screening who undergo testing by implementing an opt-out testing strategy in the EDs of 4 hospitals in the Iberian Peninsula.

## Methods

### Project design and intervention setting

A prospective, observational study was conducted after implementing opportunistic screening in the ED of four public hospitals in Spain and Portugal. The TEST model was employed in participating hospitals to promote changes in care systems and broader uptake of screening and linkage to care (LTC), as previously described^[[15–19]]^. Briefly, TEST is based on 4 main pillars: testing and linkage integrated into the normal clinical workflow, using existing infrastructure and staff to generate efficiencies (T); electronic health record (EHR) modifications (E); systemic policy change (S); and training, continuous quality enhancement and feedback by using program data to track progress, identify areas for improvement, and support staff training (T).

Screening eligibility criteria and workflows were defined in accordance with regionally applicable public health authorities’ recommendations and local epidemiological profiles, and EHR modifications were used whenever possible to automate the identification of eligible patients. To ensure that opt-out language was used, consent procedures were adjusted, and declination to participate in testing was noted in patients’ records. Following local ethics committee opinion and ECDC guidance [9], written consent forms were avoided.

Laboratory request forms and profiles were updated, and EHR systems were adjusted to automate the population of requests whenever possible. Biological specimen collection workflows were defined and integrated into regular patient pathways. To guarantee reflex testing was used, laboratory testing procedures were updated. Patient notification procedures and linkage to care workflows were defined and assigned to specific individuals.

Each site incorporated the redesigned protocols into its systems, trained its staff on the new procedures, and maintained an implementation log for monitoring and evaluating indicators that were regularly reviewed to ensure adherence to each program objective. Positive feedback loops were fostered by sharing key intervention milestones with personnel. Language and culturally sensitive patient education materials and posters were designed and visibly displayed.

Patients received appropriate clinical follow-up until discharge, regardless of test results. Information on confirmed positive individuals was automatically relayed from the laboratory to dedicated linkage to care staff by email in all participating centers. LTC staff engaged with patients to disclose the diagnosis and schedule a first visit with a specialist post-diagnosis, reattempting linkage up to 3 additional times if patients missed the visit.

### Participating centers and inclusion criteria

The EDs of four hospitals in Portugal and Spain participated in the study:

1. Hospital de Cascais Dr José de Almeida (HJA), in Cascais, Portugal, where the intervention was implemented in the adult ED which receives 98,000 visits per year. Eligible patients for HCV screening consisted of individuals aged 18–64 seeking ED care with no record of known HCV infection or tests in the past 12 months, who needed a blood draw for any reason. Data were collected between September 2018 and December 2021 (40 months).
2. Hospital Dr Nélio Mendonça (HNM) in Funchal, Portugal, where the intervention was implemented in the adult ED which receives 99,000 visits per year. Eligible patients for HCV screening consisted of individuals aged 18–70 seeking ED care with no record of known HCV infection or tests within the previous year of presentation, who needed a blood draw for any reason. Data were collected between August 2020 and January 2022 (18 months).
3. Hospital Vall d’Hebron (HUVH) in Barcelona, Spain, where the intervention was implemented in the adult ED which receives 108,000 visits per year. Eligible patients for HCV screening consisted of individuals aged 18 and over seeking ED care, with no record of known HCV infection or previous serologies in the past 3 months of presentation, who needed a blood draw for any reason. Data were collected between February 2020 and February 2022 (24 months).
4. Hospital Universitario Torrecárdenas (HUT) in Almería, Spain, where the intervention was implemented in the adult ED which receives 92,000 visits per year. Eligible patients for HCV screening consisted of individuals aged 18–70 seeking ED care, with no record of known HCV infection or previous serologies in the past 12 months, who needed a blood draw for any reason. Data were collected between August 2021 and April 2023 (20 months).

### HCV testing technology

Participants were tested for HCV Ab with ADVIA Centaur assays (Siemens Healthineers) in HJA, with Alinity s assays (Abbot Diagnostics) in HNM, with Elecsys Anti-HCV II assays (Roche Diagnostics) in HUVH, and with Liaison XL Murex HCV-Ab assays (DiaSorin) in HUT. Subsequent active infection confirmation was performed with Aptima HCV Quant Dx (Hologic) for HCV RNA in HJA, and with Cobas 6800 system (Roche) in HNM, HUVH, and HUT.

### Data analysis

The main outcomes reported were the number of HCV antibody tests performed, positive HCV antibody test results, confirmatory tests performed, and HCV viremic patients identified. Secondary outcomes included diagnosed patients’ demographic (gender, age, nationality) and clinical (liver fibrosis stage, injected drug use) characteristics, as well as the percentage of patients linked to care. All data points were analyzed descriptively to determine percentages and increases in testing rates.

## Results

### Testing volume and prevalence rates

A summary of the main results per site is shown in Table 1. In HJA and in HNM, 91 to 96% of individuals who were eligible for testing were screened after the implementation of the screening. While the two Spanish sites did not record patient declination for screening, their ratio of average monthly patients tested to the monthly patient visits to the ED (8.1–8.3%) was within the range recorded in the two Portuguese sites (7.1–13.0%). HCV testing increased many-fold across all sites, from 6.0–34.7 tests/month at baseline to 633–1164 tests/month after project implementation. Diagnosed HCV Ab seroprevalence rates varied considerably across sites, between 0.5 and 3.9%, with HCV RNA prevalence rates ranging from 0.17 to 0.73%.

**Table 1:** HCV testing volume variation before and after implementation of screening and infection rates at participating centers

**Hospital, city, country (period analyzed)**	Number of tests/month at baseline, before the intervention (mean)*	**Number of tests after the intervention (total)**	**Number of tests/month after the intervention (mean)**	**Increase in testing (%)**	**HCV Ab seroprevalence rate (%)**	**HCV RNA active infection rate (%)**
Hospital Dr. José de Almeida, Cascais, Portugal (Sept 2018–Sep 2021)	27.6	38,357	1065	3759	1.5	0.56
Hospital Dr. Nélio Mendonça, Funchal, Portugal (Aug 2020–Jan 2022)	34.7	20,954	1164	3354	0.5	0.17
Hospital Universitari Vall d’Hebron, Barcelona, Spain (Feb 2020–Feb 2022)	6.0	17,560	732	12,100	3.9	0.73
Hospital Universitario Torrecárdenas, Almería, Spain (Aug 2021–Apr 2023)	15.5	12,651	633	3984	1.7	0.35

^*^The monthly average of individuals tested within the previous 12 months prior to intervention implementation was calculated; in centers where screening was performed for less than 12 months before the intervention, a monthly average of individuals tested was calculated for the available months

*HCV Ab* hepatitis C virus antibody, *HCV RNA* hepatitis C virus ribonucleic acid

### Characteristics of the diagnosed patients

As shown in Table 2, most diagnosed patients were male. HUVH patients were older (median age of 79) than the rest of the patients from other centers, which could be attributed to that site’s broader age cohort inclusion criteria. The percentage of migrant population varied among the participating centers, with HJA having the highest rate of migrant population diagnosed with HCV (26%). We found that people who inject drugs (PWID) made up 26–43% of the diagnosed population. HUVH had the lowest linkage to care rate, at 54% of diagnosed patients, likely in relation to patients’ advanced age and comorbidities.

**Table 2:** Demographic and clinical characteristics of diagnosed patients

**Hospital, city, country (total number of HCV RNA-positive patients)**	**Age, median years**	**Males, *n* (%)**	**Migrants, *n* (%)**	**PWID, *n* (%)**	**F3–F4 advanced fibrosis, *n* (%)**	**Linkage to Care, *n* (%)**
Hospital Dr. José de Almeida, Cascais, Portugal (*n*=215)	51.3	35 (61)	15 (26)	21 (37)	12 (32)	47 (82)
Hospital Dr. Nélio Mendonça, Funchal, Portugal (*n*=51)	47.1	44 (86)	2 (4)	19 (37)	16 (33)	48 (94)
Hospital Universitari Vall d’Hebron, Barcelona, Spain (*n*=128)	79.0	59 (46)	12 (9)	33 (26)	65 (51)	69 (54)
Hospital Universitario Torrecárdenas, Almería, Spain (*n*=44)	56.0	36 (82)	6 (14)	19 (43)	7 (39)	34 (77)

Methodological note: For some patient characteristics, the denominator used for percentage calculation may be less than the total number of patients due to missing data or to patient linkage to care at other hospitals; thus, these patients are not included in the specific calculations

*HCV Ab*, hepatitis C virus antibody, *PWID* people who inject drugs

## Discussion

Our multicenter prospective study shows that the implementation of opportunistic screening for HCV in EDs is viable, does not disrupt the normal workflow of the emergency services, and is highly effective in increasing HCV diagnosis, with viremic seroprevalence rates ranging from 0.17 to 0.73%.

The identification of individuals with active HCV infection provides a critical opportunity for linkage to care, particularly when patients are in contact with the healthcare system for other conditions. Furthermore, if patients are aware of their status, they may reduce individual actions that contribute to viral transmission [20]. EDs are an important healthcare setting for efficiently testing patients for HCV. Many people at risk of hepatitis C are socially isolated. Poverty, insecure or unstructured lives, other health and social concerns, and fear of stigma and prejudice can all inhibit members of key populations from seeking and getting testing [21]. Many individuals in these groups have limited access to primary care and use the ED as their unique source of medical care. These data highlight both the need for and the importance of promoting HCV screening programs in urban EDs.

When we looked at the number of patients in our study who had HCV risk factors, we discovered that many of them had none. As a result, we considered opportunistic testing to be the most effective approach for our investigation in comparison to targeted strategies. Many studies have previously demonstrated the impact of this intervention in similar settings [22]. In the UK, a prospective, real-world study performing opt-out testing for BBVs in nine urban EDs identified 54 viral hepatitis infections in a single week, nearly half of which were newly diagnosed infections [23]. On the other hand, in Dublin, a 10-month pilot study of BBV testing in the ED in an urban hospital showed a seroprevalence of 5.1% for HCV [24]. However, the seroprevalence rates found in our study were lower than those found in previous studies in Europe (3.9% for HCV). Many factors could explain these differences. The period in which the screenings were performed might have affected the data, especially considering that our study was conducted partially during the COVID-19 pandemic. Also, the size of the EDs and local population prevalence should be considered. Although the EDs in the UK and Ireland receive roughly half the number of visits per year than the participating centers in our study, they are located in regions with higher population prevalence of HCV [23,24]. In fact, studies elsewhere in the UK found an HCV seroprevalence lower than the one reported in our investigation (0.9%) [25]. In any case, when all the data are considered together, they strongly suggest that HCV testing in urban EDs is an efficient intervention.

Several studies have evaluated the economic feasibility of performing HCV screening in EDs, establishing a cost-effectiveness threshold prevalence of viremic infection above 0.13% for such interventions [26–31]. Some studies have also analyzed the effect of opportunistic screening programs on the general population. In Spain, a study performed in a primary care setting in Valencia found an HCV RNA prevalence of 0.5% (manuscript submitted for publication). An economic evaluation of the implementation of BBV screenings in Spain showed that a prevalence of 0.13% for HCV is considered cost-effective [32], while in the UK, for screening to be considered a cost-effective intervention, the prevalence should be ≥0.26% [33]. In the case of Australia, screening was deemed cost-effective for HCV Ab prevalence rates of at least 1% [34]. Based on these data, one could extrapolate that screening for HCV would be cost-effective in the Valencia region. However, it is important to point out that there are differences that possibly limit these conclusions. Country-level epidemiology differs, affecting cost-effectiveness analyses and calculations. On the other hand, unit testing and treatment costs are lower in Spain than in the UK. In fact, HIV screening in the UK is often based on point-of-care and rapid diagnostic tests, while with the TEST model, screening is performed using laboratory-based ELISA, resulting in significant cost savings. Therefore, given both countries’ similar societal willingness to pay at average currency conversion rates, along with the lower test costs in Spain, a cost-effectiveness analysis would likely also favor universal HIV screening if calculations were adapted to Spain.

This study has some limitations. Firstly, there is significant heterogeneity between the study centers. Their linkage to care practices and eligibility criteria for screening before the application of the intervention was heterogenous, as was the degree of implementation of the TEST model. Thus, it is difficult to compare the resulting data among the different institutions. Furthermore, the period in which we analyzed the impact of the opportunistic screening program was different for each center. The COVID-19 pandemic in particular might have affected the volume of tests performed. This is particularly evident in HJA, where the intervention was implemented long before March 2020, allowing for a before-and-after comparison. Visits to EDs decreased both in Spain and Portugal after the beginning of the pandemic [35,36]. In our study, we observed a marked decline in the number of screenings conducted from March to June 2020 in HJA (972 tests/month), as compared with the pre-pandemic period (1409 tests/month from January 2019 to February 2020). Some factors may be associated with the fewer numbers of HCV tests performed during this period, including universal screening for COVID-19 at the time of admittance, changes in the ED workflow, and healthcare personnel stress levels associated with the heavy burden of the COVID-19 pandemic and fear of contracting the new virus. As long as COVID-19 remains a problem, albeit a potentially seasonal one, its impact on health services, especially in EDs, must continue to be monitored to reduce disruption of services and maintain the progress in tackling HCV transmission achieved so far. Another limitation of the lack of characterizing data on patients declining screening. However, empirical data collected with participating staff indicates the main reasons to be related to confidentiality concerns, prior knowledge of infection status, and language or health literacy barriers. Collecting data and reporting on patient treatment initiation or adherence was outside the scope of this study because of limitations related to the funding source, which is limited to screening activities occurring up to the moment of patient linkage to care post-diagnosis, to mitigate any potential conflict of interest. Another limitation is the lack of a formal cost-effectiveness analysis. Such an analysis, while critically important, is beyond the scope of this study. Moreover, as previously mentioned, recent publications have demonstrated the cost-effectiveness of non-targeted screening, although these were based on the general population and on not specific ED interventions.

## Conclusions

Our prospective multicenter study has shown that opportunistic HCV screening in EDs is feasible, does not disrupt ED activities, and is highly effective in increasing diagnosis. Considering the prevalence rates found in our program, we posit that adults seeking emergency care in high-prevalence urban settings should be considered candidate populations for robust BBV screening policies.

## Data Availability

The datasets used and/or analyzed during the current study are available from the corresponding author on reasonable request.
